# α_V_β_3_ integrin-targeted microSPECT/CT imaging of inflamed atherosclerotic plaques in mice

**DOI:** 10.1186/s13550-016-0184-9

**Published:** 2016-03-24

**Authors:** David Vancraeynest, Véronique Roelants, Caroline Bouzin, François-Xavier Hanin, Stephan Walrand, Vanesa Bol, Anne Bol, Anne-Catherine Pouleur, Agnès Pasquet, Bernhard Gerber, Philippe Lesnik, Thierry Huby, François Jamar, Jean-Louis Vanoverschelde

**Affiliations:** Pôle de Recherche Cardiovasculaire (CARD), Institut de Recherche Expérimentale et Clinique (IREC), Université Catholique de Louvain, Brussels, Belgium; Pôle d’Imagerie Médicale, Radiothérapie et Oncologie (MIRO), Institut de Recherche Expérimentale et Clinique (IREC), Université Catholique de Louvain, Brussels, Belgium; IREC Imaging Platform, Institut de Recherche Expérimentale et Clinique (IREC), Université Catholique de Louvain, Brussels, Belgium; INSERM UMR_S 1166, Integrative Biology of Atherosclerosis Team, Université Pierre et Marie Curie-Paris6 and institute of Cardiometabolism and Nutrition (ICAN), Pitié-Salpêtrière Hospital, 75013 Paris, France; Division of Cardiology, Cliniques Universitaires St-Luc, Avenue Hippocrate, 10-2881, B-1200 Brussels, Belgium

**Keywords:** α_V_β_3_ integrin, Maraciclatide, Vulnerable atherosclerotic plaque, SPECT imaging

## Abstract

**Background:**

α_V_β_3_-integrin is expressed by activated endothelial cells and macrophages in atherosclerotic plaques and may represent a valuable marker of high-risk plaques. We evaluated ^99m^Tc-maraciclatide, an integrin-specific tracer, for imaging vascular inflammation in atherosclerotic lesions in mice.

**Methods:**

Apolipoprotein E-negative (ApoE^−/−^) mice on a Western diet (*n* = 10) and normally fed adult C57BL/6 control mice (*n* = 4) were injected with ^99m^Tc-maraciclatide (51.8 ± 3.7 MBq). A blocking peptide was infused in three ApoE^−/−^ mice; this condition served as another control. After 90 min, the animals were imaged via single-photon emission computed tomography (SPECT). While maintained in the same position, the mice were transferred to computed tomography (CT) to obtain contrast-enhanced images of the aortic arch. Images from both modalities were fused, and signal was quantified in the aortic arch and in the vena cava for subtraction of blood-pool activity. The aorta was carefully dissected after imaging for gamma counting, autoradiography, and histology.

**Results:**

Tracer uptake was significantly higher in ApoE^−/−^ mice than in both groups of control mice (1.56 ± 0.33 vs. 0.82 ± 0.24 vs. 0.98 ± 0.11, respectively; *P* = 0.006). Furthermore, higher tracer activity was detected via gamma counting in the aorta of hypercholesterolemic mice than in both groups of control mice (1.52 ± 0.43 vs. 0.78 ± 0.19 vs. 0.47 ± 0.31 ^99m^Tc-maraciclatide %ID/g, respectively; *P* = 0.018). Autoradiography showed significantly higher tracer uptake in the atherosclerotic aorta than in the control aorta (*P* = 0.026). Finally, in the atherosclerotic aorta, immunostaining indicated that the integrin signal came predominantly from macrophages and was correlated with the macrophage CD68 immunomarker (*r* = 0.73).

**Conclusions:**

^99m^Tc-maraciclatide allows in vivo detection of inflamed atherosclerotic plaques in mice and may represent a non-invasive approach for identifying high-risk plaques in patients.

## Background

In the majority of cases, acute vascular events such as acute coronary syndrome or stroke are caused by the disruption of a vulnerable atherosclerotic lesion [1]. Compared to stable plaques, “rupture-prone” or “vulnerable” plaques regularly exhibit several features, such as inflammation and neoangiogenesis [2]. Identifying atherosclerotic plaques before they rupture should therefore represent a major advance in the management of atherosclerotic disease. Accordingly, invasive and non-invasive imaging techniques are under development for accurately detecting vulnerable plaques [3, 4].

The α_V_β_3_ integrin is a ubiquitous receptor that is expressed on a variety of cell types; it interacts with ligands present in the extracellular matrix or expressed on the cell surface. This integrin plays a role in diverse biological processes. α_V_β_3_ integrin is detected in situ on macrophages in early and advanced atherosclerotic lesions and could regulate macrophage functional maturation into foam cells [5]. In atherosclerotic plaques, both inflammatory cells (monocytes and macrophages) and activated endothelial cells associated with neoangiogenesis can express α_V_β_3_ integrin [6]. Therefore, α_V_β_3_ expression, which is a combined marker of both inflammation and angiogenesis, may represent a useful imaging target for assessing plaque vulnerability. Several tracers for positron emission tomography (PET) and magnetic resonance imaging (MRI) that display highly specific binding to the α_V_β_3_ integrin have been successfully tested in animal models of vascular inflammation [7–9] and in human carotid atherosclerosis [10].

^99m^Tc-maraciclatide (GE Healthcare, Amersham, UK) is a cyclic peptide that contains an arginine-glycine-aspartic acid (RGD) tripeptide sequence with a high affinity for vitronectin α_V_β_3_ integrin receptors [11]. This single-photon emission computed tomography (SPECT) tracer has been shown to localize to inflammatory infiltrate associated with angiogenesis in a murine model of hindlimb ischemia-induced angiogenesis [12, 13] and in angiogenesis induced by local IGF-1 expression after myocardial infarction [14]. More recently, maraciclatide uptake was detectable by SPECT/computed tomography (CT) in chemically injured carotid arteries in mice [15]. The potential for imaging α_V_β_3_ integrin expression in atherosclerotic plaques with ^99m^Tc-maraciclatide in spontaneous atherosclerotic mice remains unknown.

In the present study, we sought to evaluate ^99m^Tc-maraciclatide for imaging vascular inflammation by studying its in vivo uptake in the atherosclerotic mouse aorta. Uptake of the tracer in the vessel wall was validated via gamma counting and autoradiography. Specificity was confirmed with competition experiments.

## Methods

### Animal model and experimental design

**Fig. 1:**
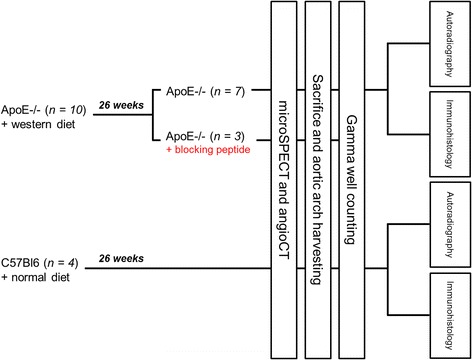
Experimental design

The study group consisted of 4- to 8-week-old male apolipoprotein E (ApoE)^−/−^ mice (*n* = 10) acquired from Taconic Europe (Lille Skensved, Denmark) and 4- to 8-week-old male C57BL/6 control mice (*n* = 4). ApoE^−/−^ mice were kept on a Western diet (1.25 % cholesterol, 16 % cocoa butter, U8220 version 97, Safe, Augy, France) for 26 weeks preceding the examinations to induce atherosclerosis. C57BL/6 mice were fed normally for the same 26 weeks. Cholesterol-fed ApoE^−/−^ mice were divided into two groups, each of which received either (1) ^99m^Tc-maraciclatide (*n* = 7) or (2) pretreatment with blocking peptide (50 μg of NC100717, unlabeled precursor, GE Healthcare, Amersham, UK) 2 min before delivery of ^99m^Tc-maraciclatide (*n* = 3). The latter group was designed to demonstrate imaging specificity. All control-diet C57BL/6 mice received ^99m^Tc-maraciclatide (*n* = 4) (Fig. 1). The experimental protocol was approved by the Institutional Animal Studies Committee of the Université Catholique de Louvain.

### MicroSPECT and CT imaging

Mice were anesthetized with 1–3 % isoflurane, and the right femoral vein was isolated for placement of a catheter (27-g butterfly needle with 12-cm polyurethane tubing, Visualsonics, Toronto, Canada) to facilitate the injection of radiotracers and contrast medium. All animals were injected intravenously with 51.8 ± 3.7 MBq of ^99m^Tc-maraciclatide, a ^99m^Tc-labeled chelated peptide conjugate containing an RGD motif targeting α_V_ integrin. Anesthesia was maintained, and each mouse was put into a sarcophagus especially designed to keep the animal in the same position throughout the experiments. A tube containing free ^99m^Tc was fixed around the sarcophagus to allow perfect fusion between SPECT images and CT images. SPECT was performed 90 min after radiotracer injection using a high-resolution small-animal imaging microSPECT system [16] (Linoview SPECT system, The Netherlands) equipped with 0.4-mm-wide slit-slat collimators. The four detectors followed four linear orbits surrounding the animal, providing linograms for a total acquisition time of 30 min with a 35 % energy window centered at 140 keV. The resulting linograms were reconstructed using the expectation maximization maximum likelihood algorithm without attenuation or resolution correction. The system spatial resolution was 0.6 mm resulting from the collimator slit width and the crystal pixel size. The sarcophagus was then transferred to a 16-detector CT system (Philips Medical System, The Netherlands). Animals were injected with a bolus infusion of CT contrast (150 μL iodinated CT contrast diluted 1:2, Iomeron 400, Bracco, UK) over 5 s, and CT imaging was performed 2 s after the start of bolus injection to precisely identify arterial structures. The following parameters were used: tube rotation speed, 420 ms; detector collimation, 16 × 0.75 mm; tube voltage, 140 kV; and effective tube current, 400 mAs.

Images from both modalities were subsequently rigidly registered using PMOD version 2.65 (PMOD Technologies, Ltd., Adliswil, Switzerland). The volume data were analyzed using the conventional transverse, coronal, and sagittal views. For quantitative analysis of tracer uptake into the plaque, a 3D region of interest (ROI) was drawn manually at the level of the aortic arch on the CT transverse images and copied to the co-registered SPECT images The totality of the arch was included in the analysis. The size of the mice vena cava being sufficient to avoid partial volume effect, a ROI in the inferior vena cava was used to determine background activity accurately. Averaged counts in each ROI were used to calculate the target to background ratio (TBR) defined as the aortic arch activity (counts/voxel) divided by the vena cava activity (counts/voxel).

### Gamma well counting

Mice were euthanized immediately after in vivo SPECT/CT. The aortic arch and the descending aorta of each animal were carefully dissected and washed in saline solution. Tissue samples were weighed. Tissue ^99m^Tc activity was measured using a gamma well counter (Packard COBRA II gamma counter) with an appropriate energy window (140 keV) and corrected for background, decay time, and tissue weight. Corrected counts were converted to μCi per milligram of tissue by using a previously determined counter efficiency. Activity was calculated as percentage of injected dose per weight (%ID/g).

### Autoradiography

Aortic samples from ApoE^−/−^ mice (*n* = 3) and from C57BL/6 control mice (*n* = 3) were exposed to an imaging plate (Fuji Imaging Plate, Fuji Photo Film Co. Ltd., Japan) immediately after gamma counting. After overnight exposure, the imaging plates were scanned with the Fuji Analyzer FLA-2000. The images were analyzed for count densities (quantification level/mm^2^) with image analysis software (AIDA Image Analyzer, Raytest, Isotopenmessgeraete GmbH, Germany) by drawing regions of interest on the aortic arch.

### Immunostaining

Aortic samples from three ApoE^−/−^ mice and from one C57BL/6 control mouse were embedded in optimum cutting temperature compound, snap-frozen, and stored at −80 °C. Histology was performed on 5 μm-thick cryosections of the aortic arch. Macrophages and endothelial cells were identified with rat monoclonal IgG (respectively: rat anti-mouse CD68, product MCA1957GA, diluted 1:50, AbD Serotec; rat anti-mouse CD31, product 550274, diluted 1:50, BD Biosciences). α_V_ integrin was identified with rabbit anti-mouse CD51 (product 210-537-R100, polyclonal, diluted 1:1000, Enzo Life Sciences). Biotinylated Alexa 568 (red) anti-rabbit or Alexa 488 (green) anti-rat IgG antibodies were used as secondary reagents. Nuclei were stained with 4′,6-diamidino-2-phenylindole. Images were acquired and digitized under high power (×63 oil-immersion objective) with a Zeiss AxioImager.z1 microscope. Signals from CD68, CD51, and CD31 were quantified with ImageJ (open-source software developed by the National Institutes of Health) and expressed as the percent positive area out of the total tissue area.

### Statistical analysis

All results are expressed as mean ± standard deviation. Continuous variables were compared among the three groups of mice using Kruskal-Wallis analysis of variance. Individual comparisons between groups were evaluated post hoc using the Mann-Whitney test with Bonferroni adjustment for multiple testing. Associations between any two variables were addressed using Pearson’s correlation. *P* < 0.05 was considered statistically significant. The statistical analyses were performed using SPSS version 1.5.0 statistical software (Chicago, IL).

## Results

**Fig. 2:**
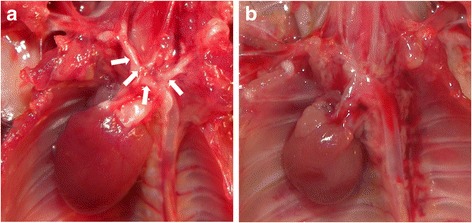
Representative images of the macroscopic appearance of the aorta of a cholesterol-fed ApoE^−/−^ mouse (**a**) and a normally fed C57BL/6 control mouse (**b**). Note the presence of atherosclerotic plaques predominantly located at the level of the aortic arch and the supra-aortic vessels of the hyperlipidemic mouse (*white arrows*); no plaque is apparent in the aorta of the control mouse

All ApoE^−/−^ mice had atherosclerotic plaques predominantly located at the level of the aortic arch and the supra-aortic vessels (Fig. 2a). As expected, no aortic plaques were found in C57BL/6 control mice (Fig. 2b).

### MicroSPECT/CT in vivo imaging of α_V_β_3_ integrin in the aortic arch

**Fig. 3:**
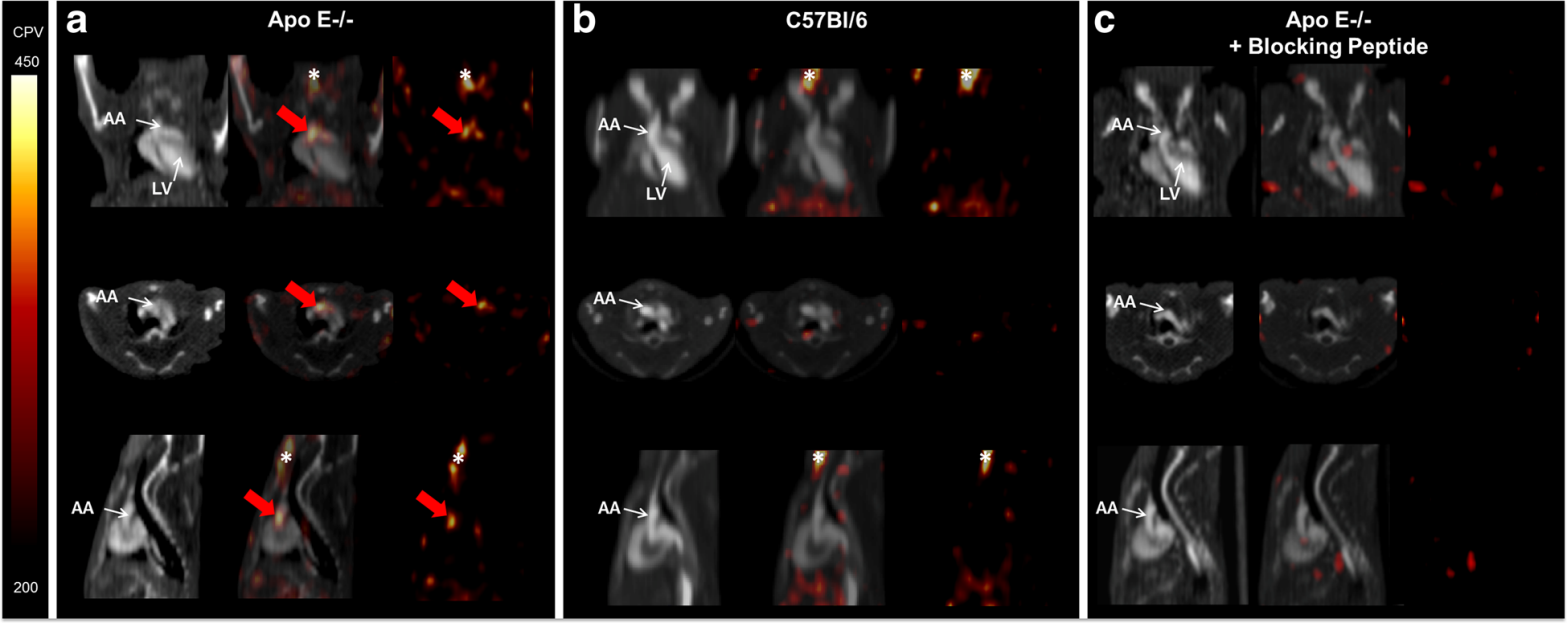
This figure shows coronal, axial, and sagittal views from CT (*left*), fused microSPECT/CT (*middle*), and SPECT (*right*) of α_V_β_3_ integrin imaging, respectively. MicroSPECT/CT allows imaging of ^99m^Tc-maraciclatide uptake (*red arrow*) into atherosclerotic plaques of ApoE^−/−^ mouse. **a** CT images, SPECT/CT overlay images, and SPECT images obtained in an ApoE^−/−^ mouse. **b** CT images, SPECT/CT overlay images, and SPECT images obtained in a C57BL/6 control mouse. **c** CT images, SPECT/CT overlay images, and SPECT images obtained in an ApoE^−/−^ mouse who received the blocking peptide. Animals were kept under gas anesthesia (1–3 % isoflurane in O_2_ at a flow rate of 2 L/min). SPECT was performed 90 min after injection of about 52 MBq of ^99m^Tc-maraciclatide. Total acquisition time was 30 min. The focal thyroid free-^99m^Tc uptake is marked by an *asterisk. LV* left ventricle, *AA* aortic arch, *CPV* counts per voxel

ApoE^−/−^ mice and control mice underwent ^99m^Tc-maraciclatide microSPECT at 30 ± 4 weeks. Increased tracer uptake was readily visible on the aortic arch of ApoE^−/−^ mice that did not receive the blocking peptide (Fig. 3a). In contrast, no signal was detected in the aortic arch of C57BL/6 control mice (Fig. 3b). As expected, no signal was visible in the aortic arch of ApoE^−/−^ mice that received the blocking peptide 2 min before ^99m^Tc-maraciclatide injection (Fig. 3c).

**Fig. 4:**
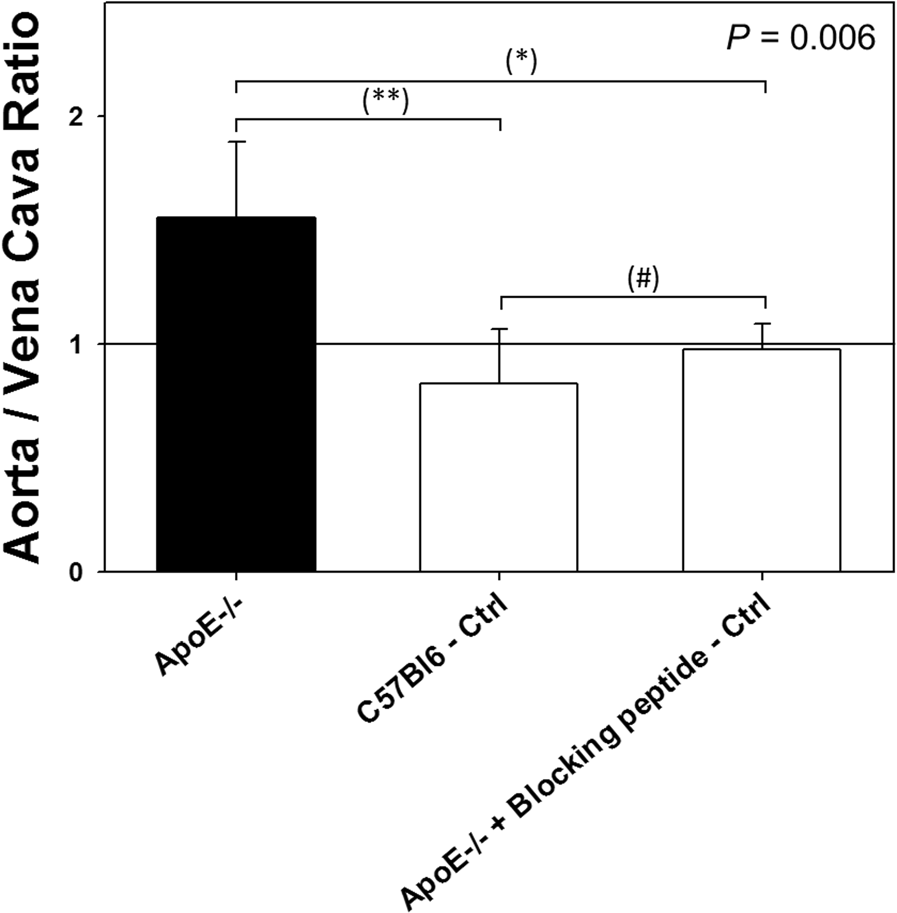
MicroSPECT-derived quantification of ^99m^Tc-maraciclatide uptake in the aortic arch of ApoE^−/−^ mice (*n* = 7), C57BL/6 control mice (*n* = 4), and ApoE^−/−^ mice who received the blocking peptide immediately before ^99m^Tc-maraciclatide injection (*n* = 3). *P* value on *top* of the graph indicates the Kruskal-Wallis test result for the three experimental groups. *Asterisks* indicate significant difference, according to the Mann-Whitney test for independent samples with Bonferroni adjustment for multiple testing; *P < 0.05, **P < 0.01. *Number sign* indicates a *P* value >0.05. *Ctrl* control

Tracer uptake was quantified in the aortic arch of all animals. Although the signal-to-noise ratio of ^99m^Tc-maraciclatide was not very high, quantitative analysis from in vivo images indicated significantly higher uptake, expressed in TBR, in the aorta of ApoE^−/−^ mice, as compared with C57BL/6 control mice and with ApoE^−/−^ mice that received the blocking peptide 2 min before ^99m^Tc-maraciclatide injection (1.56 ± 0.33 vs. 0.82 ± 0.24 vs. 0.98 ± 0.11, respectively; *P* = 0.006; Fig. 4). These data establish the specificity of tracer uptake.

### Validation of ^99m^Tc-maraciclatide in vivo uptake with gamma counting

**Fig. 5:**
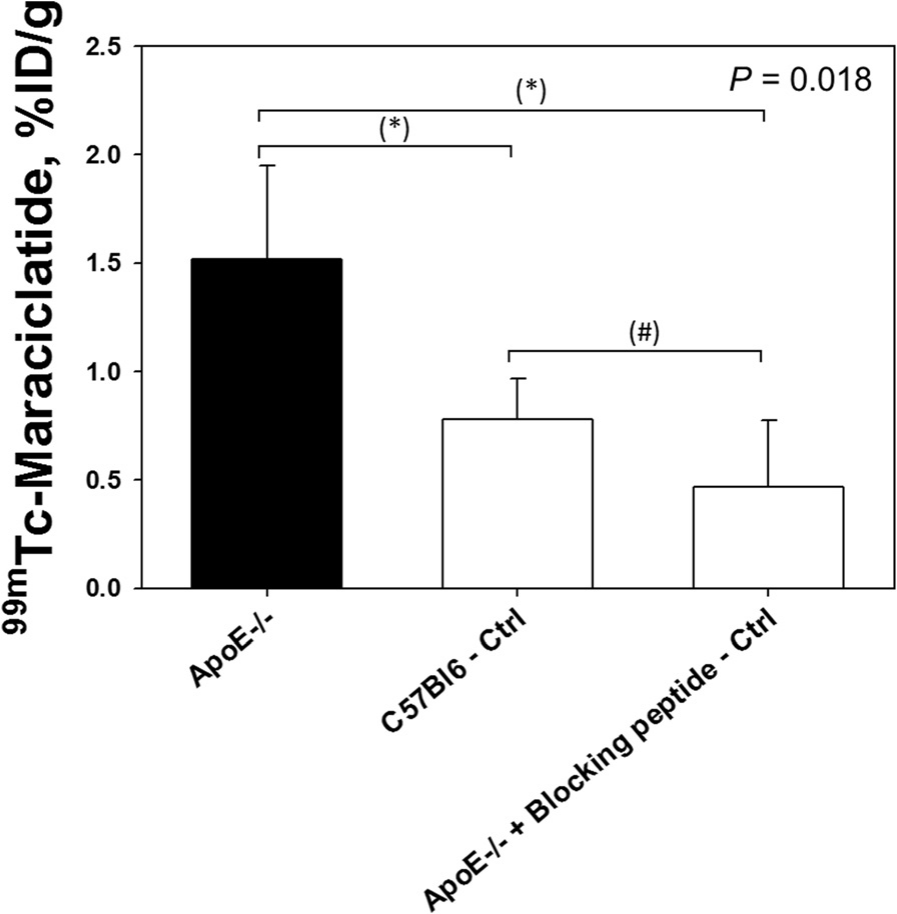
Counts obtained by gamma counting of the aortic samples of ApoE^−/−^ mice (*n* = 7), C57BL/6 control mice (*n* = 4), and ApoE^−/−^ mice who received the blocking peptide immediately before ^99m^Tc-maraciclatide injection (*n* = 3). Data are expressed as percentage of the injected dose of ^99m^Tc-maraciclatide per gram (%ID/g). *P* value on *top* of the graph indicates the Kruskal-Wallis test result for the three experimental groups. *Asterisks* indicate significant difference, according to the Mann-Whitney test for independent samples with Bonferroni adjustment for multiple testing; *P < 0.05. *Number sign* indicates a *P* value >0.05. *Ctrl* control

The significant increase in ^99m^Tc-maraciclatide uptake within the aortic arch of ApoE^−/−^ mice that was observed with microSPECT imaging in vivo was confirmed with gamma counting. ^99m^Tc-maraciclatide microSPECT uptake significantly correlated with tracer activity measured by gamma counting (*r* = 0.63, *P* = 0.038). Higher tracer activity was detected in the aortas from ApoE^−/−^ mice than in the aortas from C57BL/6 control mice and in those from ApoE^−/−^ mice that received the blocking peptide (1.52 ± 0.43 vs. 0.78 ± 0.19 vs. 0.47 ± 0.31 ^99m^Tc-maraciclatide %ID/g, respectively; *P* = 0.018; Fig. 5).

### Validation of ^99m^Tc-maraciclatide in vivo uptake with autoradiography

**Fig. 6:**
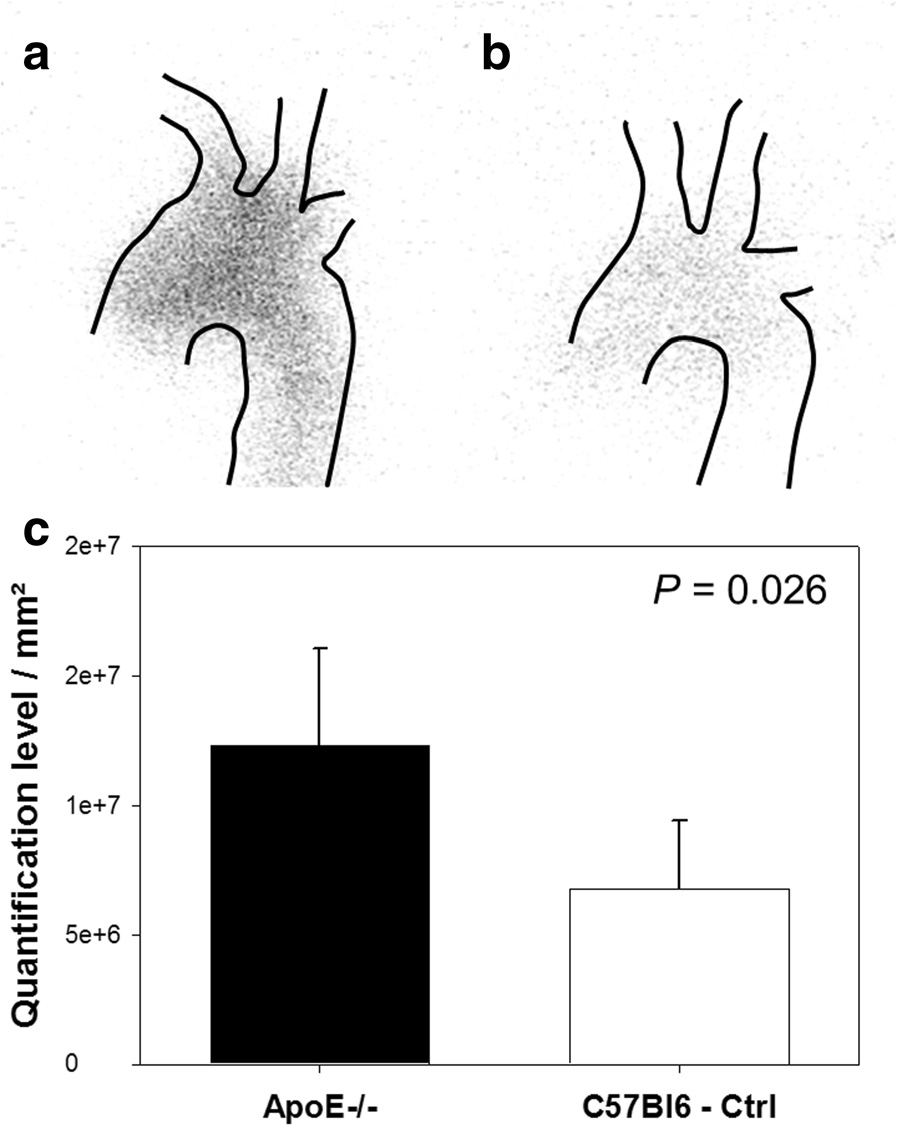
Autoradiographic analysis of ^99m^Tc-maraciclatide uptake. ^99m^Tc-maraciclatide uptake is obvious in the aorta of the ApoE^−/−^ mouse (**a**) while a minimal tracer uptake corresponding to background activity is identified in the aorta of the C57BL/6 control mouse (**b**). Higher tracer uptake in the aortas of ApoE^−/−^ mice was confirmed by drawing regions of interest around the aortic cross (**c**). *P* value on *top* of the graph indicates the Mann-Whitney test result between the two experimental groups. *Ctrl* control

To confirm the ability of ^99m^Tc-maraciclatide to detect α_V_β_3_ integrin expression in vivo, aortic arches from ApoE^−/−^ mice (*n* = 3) and from C57BL/6 control mice (*n* = 3) were harvested for autoradiography. Consistent with our microSPECT and gamma counting analyses, uptake in hyperlipidemic aortas was visibly higher than in control aortas (Fig. 6a, b). A region of interest was drawn around the entire aortic cross of the animals, and the relative autoradiographic intensity was measured. Again, the highest uptake (quantification level/mm^2^) was identified in the aortas from ApoE^−/−^ mice (*P* = 0.026; Fig. 6c).

### Immunohistology

**Fig. 7:**
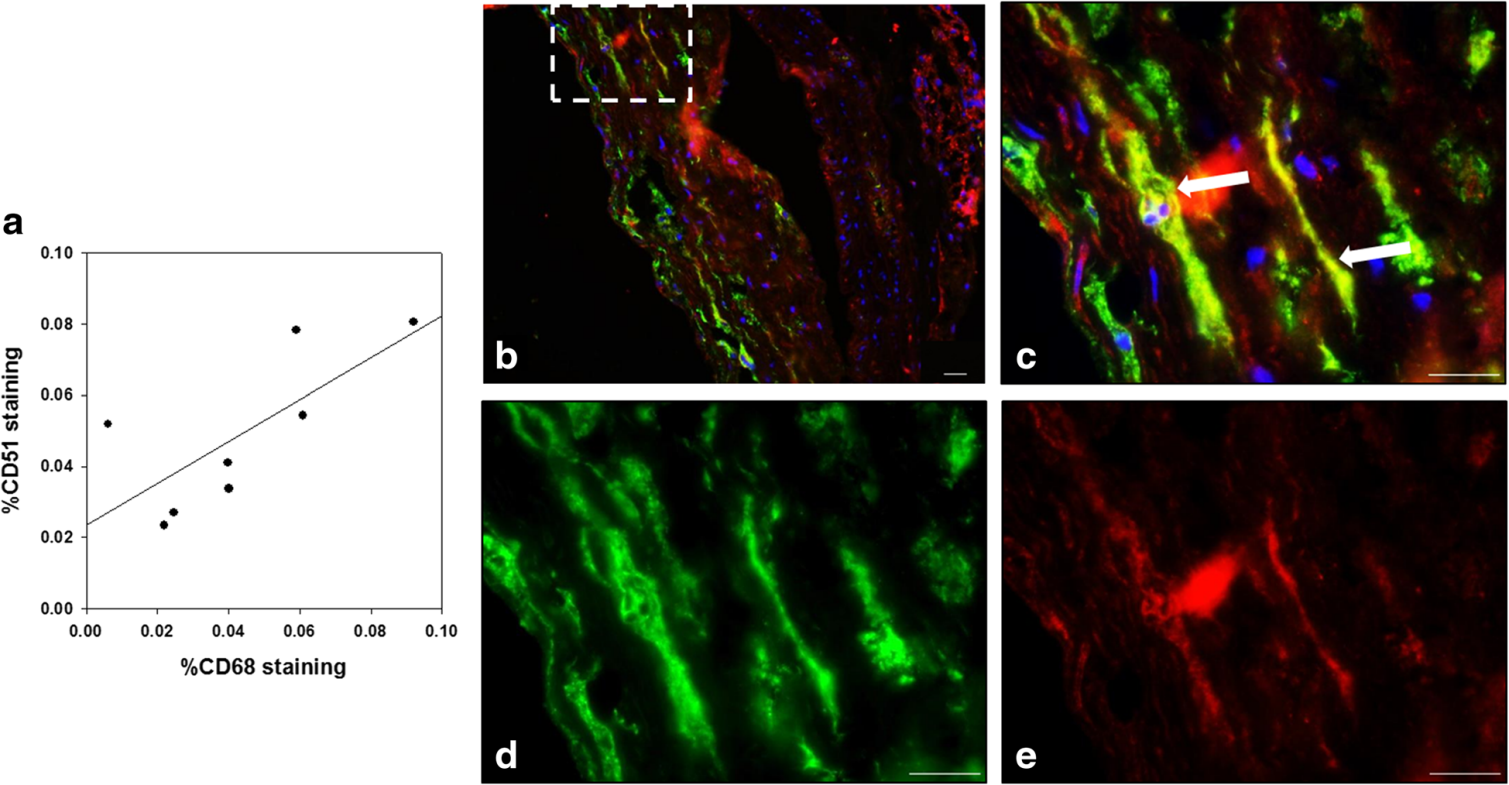
Immunostaining of inflammation and α_V_ integrin. **a** Significant correlation between percent area of CD68 staining and percent area of CD51 staining (Pearson’s *r* = 0.73, *P* = 0.04). Histopathological characterization of the aortic tissue section from an atherosclerotic animal. **b** Merged image of CD68 (macrophages in *green*) and CD51 (α_V_ integrin in *red*) co-labeling. High-power views (corresponding to the *box* in **b**) showing representative micrographs of co-labeling (**c**), macrophage (**d**), and α_V_ integrin (**e**). *Arrows* point to cells positive for both markers. Nuclei were stained with 4′,6-diamidino-2-phenylindole (*blue*) (**c**). *Scale bar* = 20 μm in **b**. *Scale bar* = 10 μm in **c**–**e**

Many types of cells in atherosclerotic plaques, including macrophages, endothelial cells, and vascular smooth muscle cells, may express α_V_ integrin. However, in our model, α_V_ integrin was mostly expressed by macrophages. Indeed, we observed a significant correlation between the patterns of CD68 and CD51 expression (*r* = 0.73, *P* = 0.04; Fig. 7a). A representative micrograph of the co-labeling of macrophages expressing CD68 and CD51 is shown in Fig. 7b–e.

**Fig. 8:**
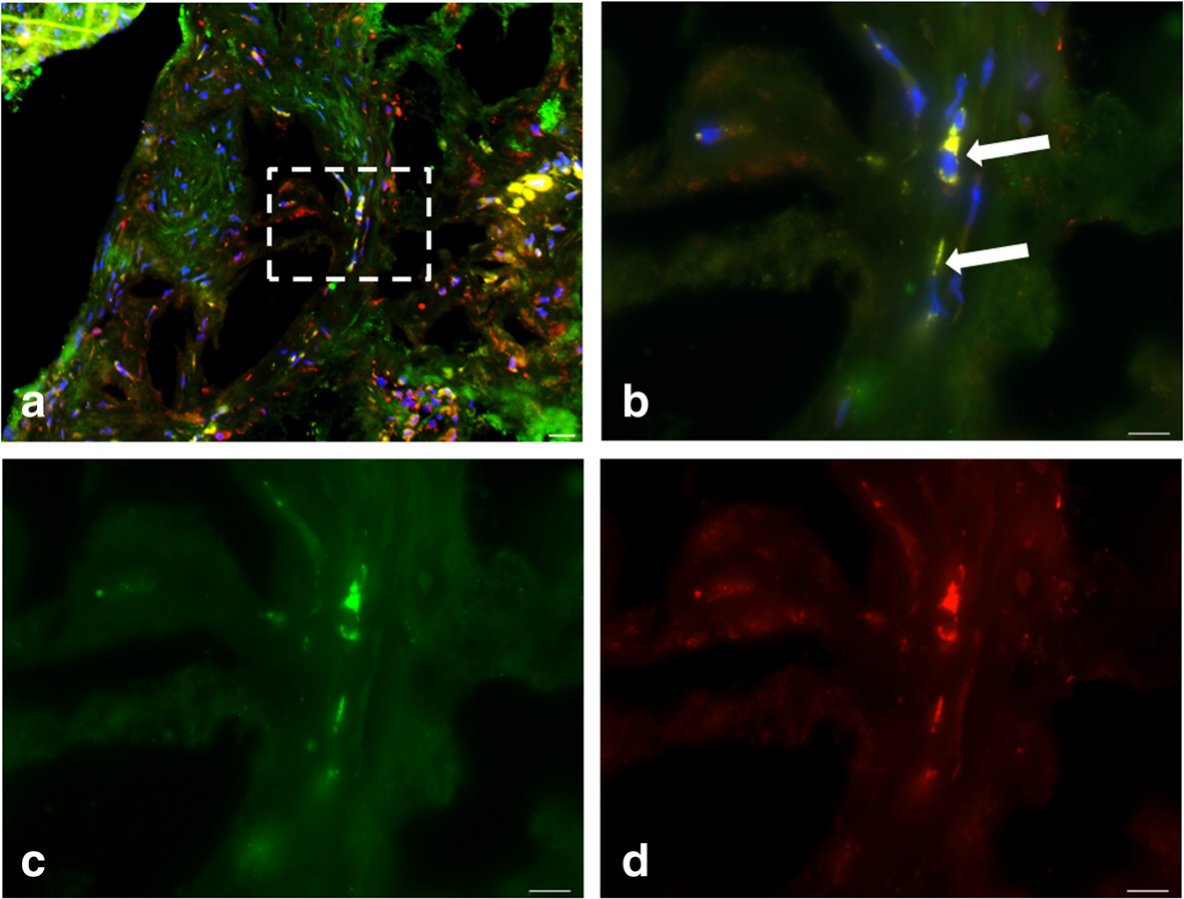
Histopathological characterization of the aortic tissue section from an atherosclerotic animal. **a** Merged image of CD31 (microvessels in *green*) and CD51 (α_V_ integrin in *red*) co-labeling. High-power views (corresponding to the *box* in **a**) showing representative micrographs of co-labeling (**b**), microvessels (**c**), and α_V_ integrin (**d**). *Arrows* point to cells positive for both markers. Nuclei were stained with 4′,6-diamidino-2-phenylindole (*blue*) (**b**). *Scale bar* = 20 μm in **a**. *Scale bar* = 10 μm in **b**–**d**

In contrast, cells expressing both CD31 and CD51 which were supposed to be neo-endothelial cells were very rarely identified in the thickness of the plaque (Fig. 8). We did not detect any correlation between percent staining for CD31 and CD51 (*r* = 0.31, *P* = 0.41).

## Discussion

Integrins belong to a group of cell-adhesion molecules; they are heterodimeric transmembrane glycoproteins that play a role in cell-cell and cell-matrix interactions [17]. Because both activated macrophages and endothelial cells can express high levels of integrin, especially α_V_β_3_ integrin, α_V_β_3_ expression represents a combined marker of inflammation and angiogenesis, which are both implicated in plaque vulnerability. α_V_β_3_ integrin binds extracellular-matrix proteins via the RGD sequence, which is present in the ^99m^Tc-maraciclatide compound. ^99m^Tc-maraciclatide has favorable kinetic properties for imaging, such as high affinity for integrin receptors, high metabolic stability in the circulation, and rapid renal excretion [11]. Our results demonstrate the potential for imaging α_V_β_3_ integrin expression with ^99m^Tc-maraciclatide and microSPECT/CT in inflamed plaques of atherosclerotic mice. We validated our results with gamma counting, autoradiography, and histology. Furthermore, competition experiments confirmed the specificity of the signal.

Atherosclerotic plaque angiogenesis has been imaged using α_V_β_3_ integrin-targeted nanoparticles and MRI [9)], and vascular inflammation has been successfully evaluated with α_V_β_3_ integrin-targeted PET [8, 10]. Here, we demonstrated that atherosclerotic lesions can be imaged with a ^99m^Tc tracer and a SPECT system, a strategy that confers several advantages. First, SPECT imaging is much more widely available and less expensive than PET or MRI. Second, the ^99m^Tc radionuclide is easier to prepare and easier to use than many PET tracers that are usually labeled with short-lived radioisotopes. Finally, like other interesting tracers (mainly PET tracers) [18, 19], ^99m^Tc-maraciclatide could be used for coronary imaging, a major area of interest. This is not the case for ^18^F-fluoro-2-deoxy-D-glucose (FDG), which is currently the most validated PET tracer for arterial inflammation imaging in humans [20]. Indeed, coronary artery imaging with that tracer is more challenging than carotid imaging, mainly due to intense FDG uptake in adjacent myocardium. Even if adequate preparation of patients allows minimal myocardial FDG uptake [21], FDG utilization in imaging of coronary wall inflammation remains to be validated; some data suggest that its utilization in coronary imaging is inappropriate [19]. Tracers like ^99m^Tc-maraciclatide that could overcome this limitation would constitute a remarkable step forward for coronary artery imaging. However, given the relative low uptake of the tracer and the spatial resolution of clinical SPECT (6.7–15.3 mm) [22], the fact that ^99m^Tc-maraciclatide is a SPECT tracer could be perceived as a limitation for its widespread use in human arterial inflammation imaging, especially for coronary artery imaging. Nevertheless, it is unlikely that in the future, vulnerable plaque identification will be based on a single imaging modality. Multimodality imaging will be probably necessary. The combined assessment of anatomical markers of high-risk plaques (soft plaques, spotty calcifications, eccentric remodeling) which can be assessed by contrast-enhanced CT with functional markers like inflammation and neoangiogenesis which can be assessed by ^99m^Tc-maraciclatide SPECT/CT could for instance represent an interesting dual approach for identifying vulnerable plaque. For this study, we used a preclinical animal scanner with a much higher spatial resolution than clinical SPECT systems [16]. Although this scanner enables sub-millimeter resolution, microscopic abdominal atherosclerosis was not visualized in our animal model. Only macroscopic plaques, such as those visually identified in the aortic arch (Fig. 2), were imaged with ^99m^Tc-maraciclatide, suggesting that a “critical mass” is needed for plaque imaging. Whether the combined use of ^99m^Tc-maraciclatide and clinical SPECT scanners will enable the detection of inflamed atherosclerotic plaques in humans remains unknown. However, cooperation between researchers and manufacturers will ensure that SPECT/CT becomes a more accurate and reliable tool in the next decade.

The plaques in our ApoE-deficient mice are “human-like,” as they are very similar to plaques observed in patients [23]. Furthermore, in ApoE^−/−^ mice like those we used in this work, plaques which are present at the origin of the great vessels of the neck and on which we concentrated in this study (Fig. 2) demonstrate features likely to be the “murine parallel” of those in vulnerable human plaques [24]. We therefore believe that this tracer may be of interest for detecting high-risk plaques in humans and perhaps also in tracking the effect of aggressive pharmacological treatment, like high dose of statins. Furthermore, this tracer has been approved for human use; its safety and tolerance have already been successfully tested in patients with breast cancer [25]. Therefore, it would be interesting to move from preclinical animal studies to clinical studies in order to test this tracer in the field of human atherosclerosis.

We detected a strong correlation between immunostaining for α_V_ integrin (CD51) and for macrophages (CD68); in contrast, co-staining for α_V_ integrin and neovessels (CD31) identified very few cells that were positive for both immunomakers. This finding is similar to observations reported by previous studies [8, 15, 26]. It also suggests that the ^99m^Tc-maraciclatide signal largely results from inflammatory infiltrate rather than from neoangiogenesis per se. Inflammation plays a key role in atherosclerosis and is linked to plaque vulnerability. The presence of inflammation in the arterial wall of large arteries measured by FDG-PET/CT has been shown to predict acute clinical events [27]. ^99m^Tc-maraciclatide could thus help to refine the clinician’s ability to stratify patient risk for acute events such as stroke, sudden cardiac death, or acute coronary syndromes.

Some limitations must be acknowledged for this study. First, the number of animals is small. Since it was unknown whether reliable SPECT signal could be obtained from the atherosclerotic lesions of mice, this study was designed as a “feasibility study.” Nonetheless, tracer uptake was clearly detected in the aortic arch of atherosclerotic mice in comparison with control animals. Furthermore, the imaging results were validated with several other techniques, including gamma counting and autoradiography, and blocking with excess unlabeled precursor confirmed the specificity of the SPECT signal. Finally, the number of animals used in this study is similar to that used in other comparable studies [8, 26]. As a second limitation, we did not measure mRNA expression of the genes encoding CD68, CD31, or CD51 in mouse aortas via quantitative reverse transcription polymerase chain reaction. Nevertheless, we obtained convincing images with immunohistology, and the quantification of the various markers revealed a clear relationship between integrin expression and macrophages. The co-expression of integrin and CD31 was clearly weaker. These results are consistent with those obtained by Yoo et al [26].

## Conclusions

Here, we have demonstrated that ^99m^Tc-maraciclatide SPECT/CT specifically detects α_V_β_3_ integrin expression in inflamed atherosclerotic plaques in ApoE^−/−^ mice. This tracer may thus represent an interesting non-invasive approach for identifying high-risk plaques in human patients and for monitoring their global risk for presenting acute vascular events. Its clinical value must be defined by future prospective human studies.
